# Decreased expression of NAT10 in peripheral blood mononuclear cells from new-onset ankylosing spondylitis and its clinical significance

**DOI:** 10.1186/s13075-023-03250-0

**Published:** 2024-01-02

**Authors:** Qing Luo, Juxiang Zhu, Shiqian Wang, Peng fu, Biqi Fu, Zikun Huang, Junming Li

**Affiliations:** 1 Department of Clinical Laboratory, The First Affiliated Hospital, Jiangxi Medical College, Nanchang University, Nanchang, Jiangxi 330006 China; 2 Institute of Infection and Immunity, Jiangxi Medical College, Nanchang University, Nanchang, Jiangxi 330006 China; 3 Nanchang Key Laboratory of Diagnosis of Infectious Diseases, Nanchang, Jiangxi 330006 China; 4 Jiangxi Medical College, Nanchang University, Nanchang, Jiangxi 330006 China; 5 Department of Rheumatology, The First Affiliated Hospital, Jiangxi Medical College, Nanchang University, Nanchang, Jiangxi 330006 China; 6 Medical Center of Burn Plastic and Wound Repair, The First Affiliated Hospital, Jiangxi Medical College, Nanchang University, Jiangxi, 330006 China

**Keywords:** NAT10, Ankylosing spondylitis, Diagnosis

## Abstract

**Background:**

NAT10 is the firstly recognized RNA acetyltransferase that participates in multiple cellular biological processes and human disease. However, the role of N-acetyltransferase 10 (NAT10) in ankylosing spondylitis (AS) is still poorly elaborated.

**Methods:**

Fifty-six patients with New-Onset AS, 52 healthy controls (HC), 20 patients with rheumatoid arthritis (RA) and 16 patients with systemic lupus erythematosus (SLE) were recruited from The First Afliated Hospital of Nanchang University, and their clinical characteristics were recorded. The expression level of *NAT10* in peripheral blood mononuclear cell (PBMC) was examined using reverse transcription-quantitative PCR analysis. The correlations between the expression level of *NAT10* in the New-Onset AS patients and disease activity of AS were examined, and receiver operating characteristic (ROC) curves were built to evaluate predictive value in AS. Univariate analysis and multivariate regression analysis were used to analyze the risk factors and construct predictive model.

**Results:**

The mRNA expressions of *NAT10* in PBMC from new-onset AS patients were significantly low and there were negative correlation between mRNA *NAT10* and ASDAS-CRP, BASDIA in new-onset AS patients. ROC analysis suggested that mRNA *NAT10* has value in distinguishing new-onset AS patients from HC, RA and SLE. Furthermore, a novel predictive model based on mRNA *NAT10* and neutrophil percentages (N%) was constructed for distinguishing new-onset AS patients from HC (AUC = 0.880, sensitivity = 84.62%, specificity = 76.92%) and the predictive model correlated with the activity of new-onset AS. Furthermore, the predictive model could distinguish new-onset AS patients from RA and SLE (AUC = 0.661, sensitivity = 90.38%, specificity = 47.22%). Moreover, the potential predictive value of the combination of predictive model-HLA-B27 for AS vs. HC with a sensitivity of 92.86% (39/42), a specificity of 100.00% (52/52) and an accuracy of 96.81% (91/94) was superior to that of HLA-B27, which in turn had a sensitivity of 84.44% (38/45), a specificity of 100.00% (52/52) and an accuracy of 92.78% (90/97).

**Conclusion:**

The present study suggested that the decreased mRNA *NAT10* may play a role in AS pathogenesis and predictive model based on mRNA *NAT10* and N% act as bioindicator for forecast and progression of diseases.

## Introduction

Ankylosing spondylitis (AS) is a type of rheumatic disease that leads to systemic inflammation and osteogenesis, and is a highly disabling and destructive type of arthritis [1]. AS attack mostly young people and affect 0.09%-0.3% of the global population [2]. Although the AS pathogenesis is mainly involved in the combined effects of environmental triggers, infection, genetic susceptibility, and immune disorders, the concrete pathogenesis is still unclear [2]. Due to the limited understanding of disease pathogenesis, there is no diagnostic test specific to AS and there is still a lack of specific therapeutic targets for AS, which usually cause a delay in onset diagnosis for AS and cannot get effective treatment in time [3, 4].

N4-acetylcytidine (ac4C) is a highly conserved modification of RNA. N-acetyltransferase 10 (NAT10) increased the formation of ac4C on Erna, Erna, and mRNA, thereby maintaining the accuracy of protein translation and stabilizing the mRNA [5]. NAT10 involves in multiple biological function via its acetyltransferase activity, such as apoptosis, pyroptosis, proliferation, metastasis, and autophagy [6–11]. Interestingly, the prognostic and immunological role of NAT10 have been observed in multiple tumors [12]. Remarkably, aberrant NAT10 and the ac4C levels also contributes to infectious diseases including HIV and influenza A virus infection [13, 14]. Moreover, several studies also show that level of NAT10 are associated with inflammatory responses and systemic lupus erythematosus (SLE) [15, 16]. All these studies demonstrate the multi-functionality of NAT10. However, the role of NAT10 in AS pathogenesis is still poorly elaborated.

In the present study, we firstly determined the mRNA expression of *NAT10* in peripheral blood mononuclear cell (PBMC) from patients with new onset AS and evaluated its clinical significance. We demonstrated that the mRNA level of *NAT10* in PBMC from patients with new onset AS was significantly decreased than that in healthy controls (HC), rheumatoid arthritis (RA) and SLE, and decreased *NAT10* negatively correlated with the activity of AS. Further research showed that the predictive model based on *NAT10* and neutrophil percentages (N%) exhibited a better predictive value for distinguishing patients with new-onset AS from HC with the area under the curve (AUC) = 0.880 and the predictive model positively correlated with the activity of AS. And, the predictive model showed a certain predictive value for distinguishing patients with new-onset AS from RA + SLE. Furthermore, the combination of predictive model (mRNA *NAT10*-N%) and HLA-B27 could further improve the diagnostic value. Thus, our findings have proved that NAT10 involved in the pathogenesis and was an independent predictive biomarker of new-onset AS.

## Materials and methods

### Sample subjects

Potential sample subjects including AS who fulfilled the modified New York 1984 criteria for AS [17], and age or sex-matched healthy control (HC) without autoimmune diseases and free of other inflammatory conditions for this study were consecutively enrolled from the First Affiliated Hospital of Nanchang University. Those AS patients with other inflammatory, autoimmune, or hormonal diseases, cancers, or mental disorders, were excluded. All AS patients were new-onset that was diagnosed for the first time and had not yet used immunosuppressive agents or corticosteroids prior to recruitment. In addition, RA patients fulfilled the revised ACR criteria for RA [18] and SLE patients fulfilled the revised ACR criteria for SLE [19] were recruited from the First Affiliated Hospital of Nanchang University as autoimmune disease control. All study protocols complied with the principles outlined in the Declaration of Helsinki and was approved by the Ethics Committee of The First Affiliated Hospital of Nanchang University (approval no. (2023) CDYFYYLK(01–057)).

### Collection of peripheral blood mononuclear cell samples and RNA extraction

Over the course of the study, the trained staff collected all subjects and their clinical and laboratorial information. To isolate peripheral blood mononuclear cell (PBMC), fasting blood (3 mL) was collected from the elbow vein into EDTA-coated tubes and then the PBMC was isolated using previously-reported protocols [20]. Thereafter, the determination of cell concentrations in each isolated sample, 2 × 10^6^ PBMC/patient were incubated with a 0.75 ml TRIzol reagent (Invitrogen Bio, Waltham, MA, United States) and total RNA was then extracted according to manufacturer protocols. RNA integrity and quantity from AS patients and HC were determined (using A260/A280 and A260/A230 ratios) by a NanoDrop ND-1000 spectrophotometer (Invitrogen Bio, Waltham, MA, United States). The final isolates were each then stored at -80℃ until PCR analyses.

### Reverse transcription-quantitative PCR analysis

The cDNA samples were acquired from 1 g total RNA/isolate by a reverse transcription reaction using a PrimeScript RT kit (Takara Bio Inc., Kyoto, Japan).

Thereafter, the product was used as a template for PCR in an ABI 7500 Real-time PCR System (Invitrogen, California, USA) that employed SYBR Premix Ex TaqTM II (Takara Bio Inc., Kyoto, Japan). And the protocol for the PCR assays was as follows: initial denaturation step at 95℃for 5 min, followed by 40 cycles of 15 s at 95℃ (denaturation), 1 min at 60℃ (annealing and elongation), and 2 min at 72℃ (final extension). The primers of *NAT10* (F: 5’GGGATTGGCCTGCAGCATA3’, R: 5’GGCTCCATGACCACATCCTT3’) used in the present study were designed by Primers 5 software, verified by primer-BLAST and synthesized by Shanghai Shenggong. In each subject, *GADPH* (F: 5’TGCACCACCAACTGCTTAGC3’, R: 5’GGCATGGACTGTGGTCATGAG3’) was used as an internal control. For all subjects, relative *NAT10* expression was derived using the 2^−ΔΔCt^ comparative threshold cycle method [8, 21].

### Clinical assessments and laboratory indexes

Blood samples from the EDTA tubes were used to detect blood routine parameters of AS patients and HC by a Sysmex XE-2100 analyzer (Sysmex Bio, Kobe, Japan), including the white blood cell counts (WBC), lymphocyte counts (L) and percentages (L%), monocyte counts (M) and percentages (M%), neutrophil counts (N) and percentages (N%), red blood cell counts (RBC), hemoglobin (HGB), hematocrit (HCT), platelet count (PLT), plateletcrit (PCT), mean platelet volume (MPV), and platelet distribution width (PDW). Erythrocyte sedimentation rate (ESR) was examined by an Automatic ESR analyzer TEST1 system (ALIFAX Bio, Udine, Italy). The IMMUNE 800 system (Beckman Coulter, San Jose, CA, United States) was used to evaluate serum levels of CRP from AS patients. The negative or positive results of human leukocyte antigen-B27 (HLA-B27) were acquired using HLA-B27 nucleic acid detection kit (fluorescent PCR method) (Suzhou Tianlong Biotechnology Co., LTD, China) on ABI 7300 Real-time PCR System (Invitrogen, California, USA). The disease activity of AS was evaluated by the AS Disease Activity Score-C reaction protein (ASDAS-CRP), the Bath AS Disease Activity Index (BASDAI), and the Bath AS Functional Index (BASFI) [22, 23].

### Statistical analysis

All data are expressed in terms of means ± SE. Statistical analysis was performed using GraphPad Prism (version 5.0; GraphPad Software, Inc.) and SPSS (version 16.0; SPSS, Inc.). A Student’s t-test or a Mann–Whitney U-test was used to compare the differences in *NAT10* expression and blood routine parameters between groups according to the normality. The Spearman method was used for correlation analysis. Risk factors analysis was performed using univariate analysis and multivariate regression analysis. Receiver operating characteristic (ROC) curves were performed to evaluate the predictive efficiency of *NAT10*, blood routine parameters and predictive model. A two-sided *P* < 0.05 was considered to indicate statistical significance.

## Results

### Characteristics of the study subjects

A total of 144 subjects were recruited in the present study from Jul 2020 to Nov 2023, including 56 patients with new-onset AS, 52 HC, 20 RA and 16 SLE subjects. The demographic characteristics of new-onset AS, HC and RA + SLE subjects are provided in Table 1. No significant differences were noted between patients with new-onset AS and HC subjects regarding age or sex. Autoimmune disease control (Patients with SLE and RA) and AS were not age or sex-matched due to the difference in age or sex of onset of the these diseases (the incidence of RA was high among women 50–60 years of age and the incidence of SLE was high among women 20–40 years of age, while that of AS was high among men of 10–40 years of age). Moreover, the WBC, PLT, PDW, N and N% were significantly increased in the new-onset AS as compared with those in HC subjects, while the HGB, HCT, MPV, L, L% and M% were significantly decreased (all *P* < 0.0500; Table 1). When compared to RA + SLE, the RBC, HGB, HCT, L and L% were significantly increased in the new-onset AS, while the PDW and N% were significantly decreased (all *P* < 0.0500; Table 1).

**Table 1:** Demographic characteristics of new-onset AS, HC and RA + SLE subjects

Demographic characteristics	HC	New-onset AS	RA + SLE
Age (years)	32.56 ± 9.37	30.95 ± 10.55	50.67 ± 15.13^#^
Sex (male/female)	35/17	38/18	4/32^#^
ASDAS-CRP		2.87 ± 1.20	
BASDAI		4.48 ± 1.80	
BASFI		4.04 ± 1.51	
HLA-B27 positive/total	0/52	38/45	
CRP (mg/l)		18.86 ± 31.68	
ESR (mm/h)		20.87 ± 21.93	
WBC (10^9^/l)	6.39 ± 1.15	7.27 ± 1.63^*^	7.26 ± 3.24
RBC (10^12^/l)	5.00 ± 0.45	4.87 ± 0.72	4.17 ± 0.47^#^
HGB (g/l)	150.65 ± 12.34	139.5 ± 16.28^*^	119.61 ± 17.88^#^
HCT (l/l)	0.45 ± 0.04	0.43 ± 0.05^*^	0.38 ± 0.05^#^
PLT (10^9^/l)	243.02 ± 42.30	288.73 ± 80.33^*^	267.63 ± 114.89
MPV (fl)	10.55 ± 0.82	9.78 ± 1.07^*^	10.12 ± 1.36
PCT (%)	0.25 ± 0.04	0.28 ± 0.07	0.26 ± 0.09
PDW (fl)	12.66 ± 1.69	14.43 ± 2.40^*^	15.93 ± 0.89^#^
L (10^9^/l)	2.24 ± 0.50	1.90 ± 0.48^*^	1.39 ± 0.67^#^
L (%)	35.50 ± 7.25	26.81 ± 5.92^*^	22.71 ± 7.02^#^
M (10^9^/l)	0.45 ± 0.15	0.44 ± 0.13	0.43 ± 0.18
M (%)	6.70 ± 1.76	6.18 ± 1.72^*^	6.57 ± 2.34
N (10^9^/l)	3.54 ± 0.91	4.78 ± 1.42^*^	5.33 ± 2.76
N (%)	55.02 ± 7.32	65.02 ± 7.17^*^	71.26 ± 8.64^#^

*AS* ankylosing spondylitis, *ASDAS* AS Disease Activity Score, *BASDAI* Bath AS Disease Activity Index, *BASFI* Bath AS Functional Index, *CRP* C reaction protein, *ESR* erythrocyte sedimentation rate, *HC* healthy control, *HCT* hematocrit, *HGB* hemoglobin, *HLA-B27* human leukocyte antigen-B27, *L* lymphocyte counts, *L%* lymphocyte percentages, *M* monocyte counts, *M%* monocyte percentages, *MPV* mean platelet volume, *N* neutrophils counts, *N%* neutrophil percentages, *PCT* plateletcrit, *PDW* platelet distribution width, *PLT* platelet count, *RA* rheumatoid arthritis, *RBC* red blood cell counts, *SLE* systemic lupus erythematosus, *WBC* white blood cell counts

^*^P < 0.05, AS compared to HC

^#^*P* < 0.05, AS compared to RA + SLE

### The expression of mRNA *NAT10* in peripheral blood mononuclear cell from patients with new-onset AS was low

**Fig. 1:**
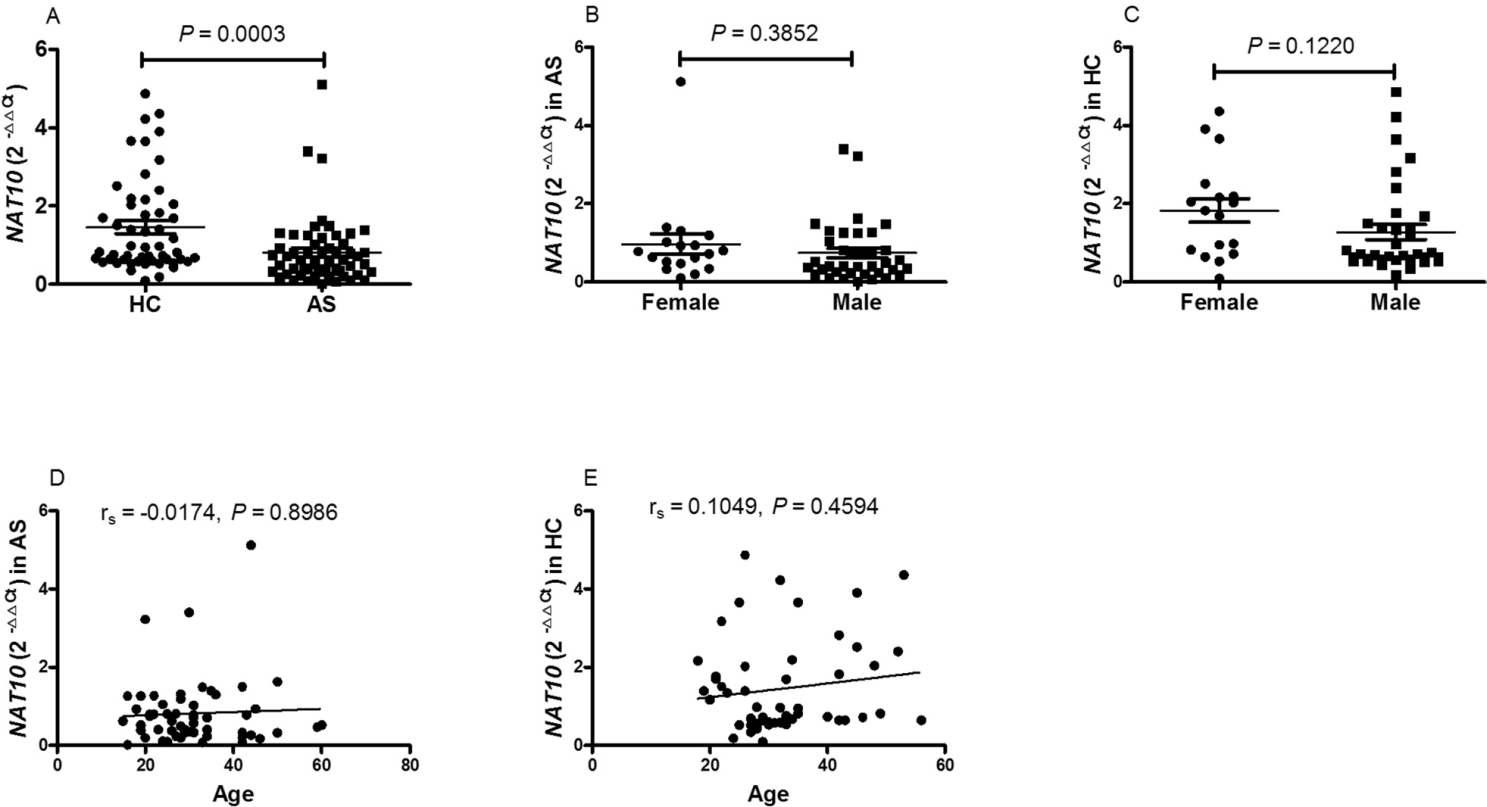
Expression of mRNA *NAT 10* in HC, AS and their association with sex, age. **A** AS patients had decreased mRNA *NAT10* compared with HC. **B** The expression of mRNA *NAT10* was similar in female and male from AS patients. **C** The expression of mRNA *NAT10* was similar in female and male from HC. **D** The expression of mRNA *NAT10* was not associated with age in AS patients. **E** The expression of mRNA *NAT10* was not associated with age in HC. AS: ankylosing spondylitis, HC: healthy control, *NAT10*: N-acetyltransferase 10

The mRNA expression level of *NAT10* in PBMC was first evaluated in patients with new-onset AS and the HC subjects using qRT-PCR. A significantly lower expression of *NAT10* was observed in the patient group with new-onset AS compared to the HC group (*P* = 0.0003; Fig. 1A). No association was noted between mRNA expression level of *NAT10* and age or sex in patients with new-onset AS or HC subjects (all *P* > 0.0500; Fig. 1B-E).

### Decreased mRNA *NAT10* expression in peripheral blood mononuclear cell correlates with disease activity of new-onset AS

**Fig. 2:**
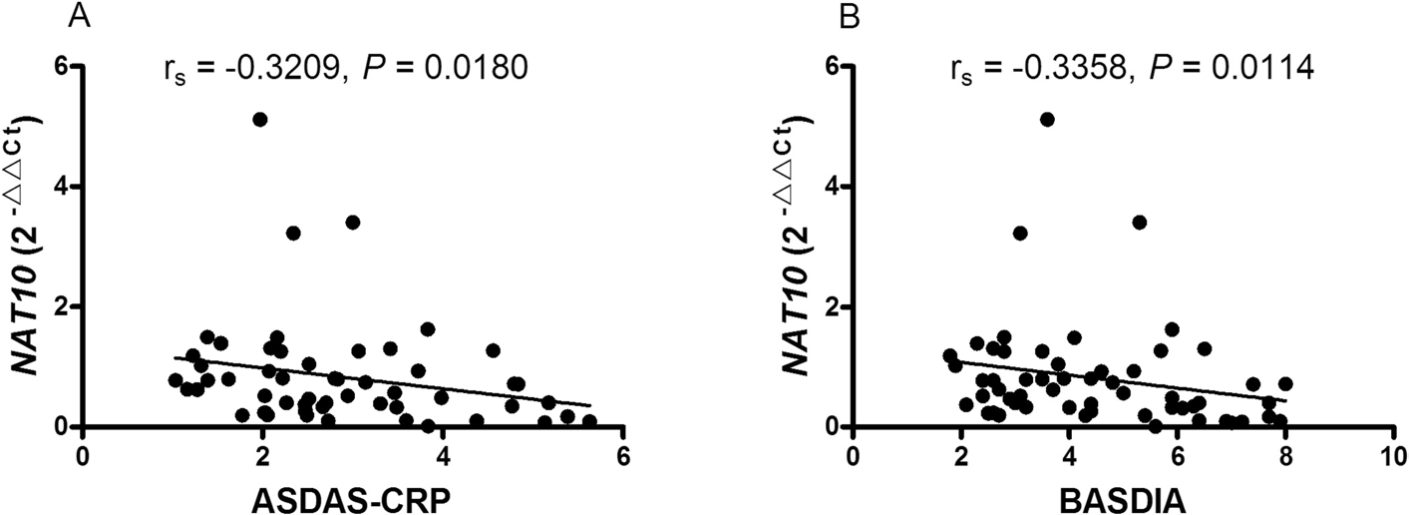
Correlation between the expression of mRNA *NAT10* in PBMC from AS patients with disease activity. **A** The expression of mRNA NAT10 in PBMC negatively correlated with ASDAS-CRP. **B** The expression of mRNA NAT10 in PBMC negatively correlated with BASDIA. AS: ankylosing spondylitis, ASDAS-CRP: AS Disease Activity Score-C reaction protein, BASDAI: Bath AS Disease Activity Index, *NAT10*: N-acetyltransferase 10, PBMC: peripheral blood mononuclear cell

To explore whether the mRNA expression level of *NAT10* in PBMC from patients with new-onset AS could be used to assess disease activity, Spearman correlation test was used to investigate the relationship between the mRNA expression level of *NAT10* and clinical characteristics including ASDAS-CRP, BASDAI, BASFI, CRP, ESR, ECR, WBC, RBC, HGB, HCT, PLT, MPV, PCT, PDW, L, L%, M, M%, N, N%. The decreased mRNA expression level of *NAT10* in PBMC from patients with new-onset AS was found to correlate with ASDAS-CRP (r_s_ = -0.3209, *P* = 0.0180; Fig. 2A), BASDIA (r_s_ = -0.3358, *P* = 0.0114; Fig. 2B). However, the decreased mRNA expression level of *NAT10* in PBMC from patients with new-onset AS did not correlate with other clinical characteristics that indicates the activity of the disease (data no shown).

**Fig. 3:**
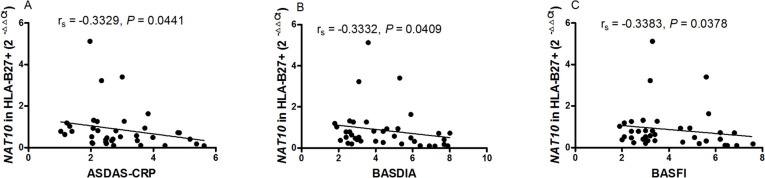
Correlation between the expression of mRNA *NAT10* in PBMC from HLA-B27 positive AS patients with disease activity. **A** The expression of mRNA NAT10 in PBMC from HLA-B27 positive AS patients negatively correlated with ASDAS-CRP. **B** The expression of mRNA NAT10 in PBMC from HLA-B27 positive AS patients negatively correlated with BASDIA. **C** The expression of mRNA NAT10 in PBMC from HLA-B27 positive AS patients negatively correlated with BASFI. AS: ankylosing spondylitis, ASDAS-CRP: AS Disease Activity Score-C reaction protein, BASDAI: Bath AS Disease Activity Index, BASFI: Bath AS Functional Index, HLA-B27: human leukocyte antigen-B27, NAT10: N-acetyltransferase 10, PBMC: peripheral blood mononuclear cell

HLA-B27 is the primary laboratory index to diagnose AS. Among the 56 patients with AS, 45 patients were tested for HLA-B27 and 38 patients were positive. Then, we explored the the association between the decreased mRNA expression level of *NAT10* in PBMC from patients with new-onset AS and HLA-B27. However, no difference was found between HLA-B27 positive patients with new-onset AS and HLA-B27 negative patients with new-onset AS in the mRNA expression level of *NAT10* (data no shown). Furthermore, the relationship between the mRNA expression level of *NAT10* in HLA-B27 positive patients with new-onset AS and the above clinical characteristics was determined. The decreased mRNA expression level of *NAT10* in PBMC from HLA-B27 positive patients with new-onset AS was found to correlate with ASDAS-CRP (r_s_ = -0.3329, *P* = 0.0441; Fig. 3A), BASDIA (r_s_ = -0.3332, *P* = 0.0409; Fig. 3B), BASFI (r_s_ = -0.3383, *P* = 0.0378; Fig. 3C).

### Using *NAT10* and routine laboratory indicators for predicting patients with new-onset AS from HC

**Fig. 4:**
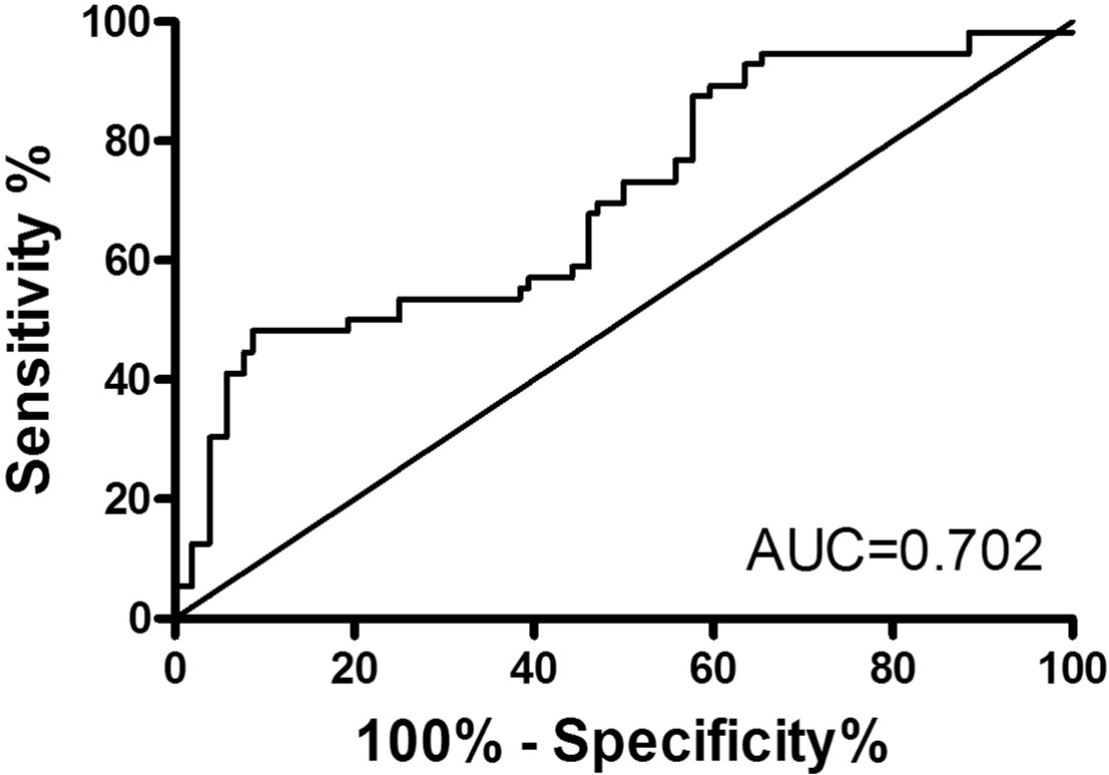
ROC analysis for the predictive value of the expression of mRNA *NAT10*. NAT10: N-acetyltransferase 10, ROC: receiver operating characteristic

Therefore, a ROC curve was drawn to assess the potential predictive value of the mRNA expression level of *NAT10* in PBMC for new-onset AS, and the values showed that the area under the ROC curve (AUC) for distinguishing new-onset AS from HC was up to 0.702 [95% CI = 0.604–0.800; *P* = 0.0003], with a cutoff value of < 0.5212, a sensitivity of 48.21%, and a specificity of 90.38% (Fig. 4).

**Fig. 5:**
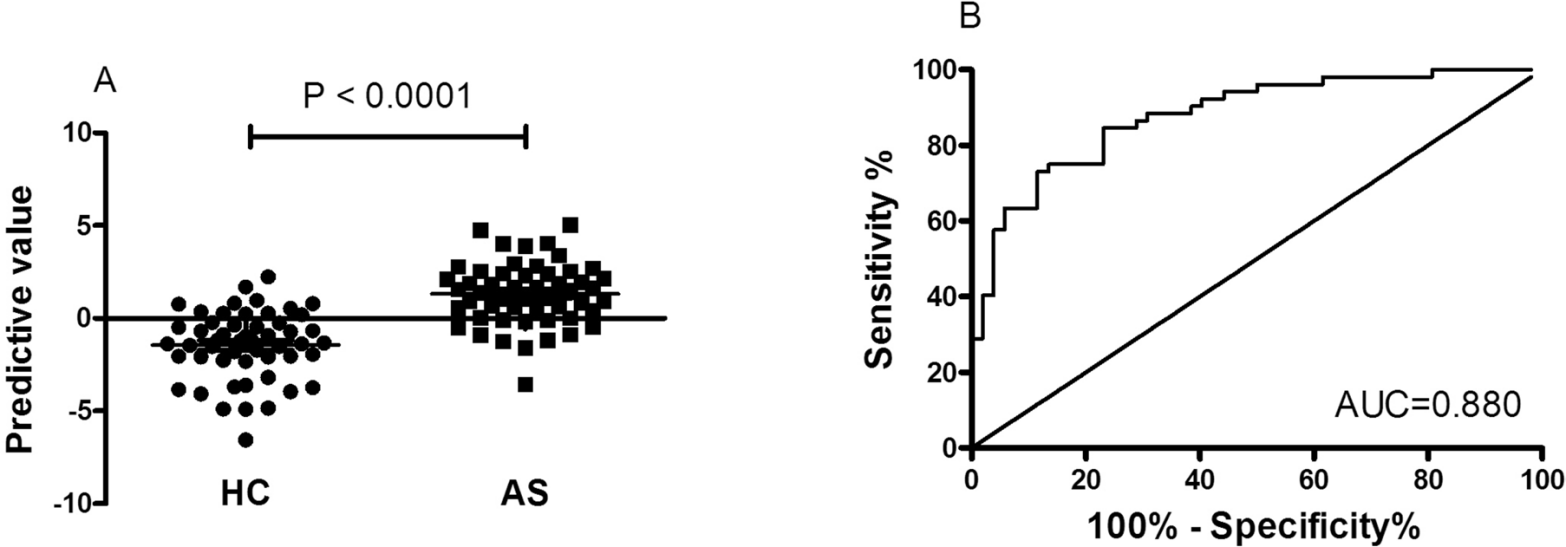
The predictive value based mRNA *NAT10* and N%, and the ROC analysis. **A** The predictive value between AS patients and HC. **B** ROC analysis of predictive model. NAT10: N-acetyltransferase 10, N%: neutrophil percentages, ROC: receiver operating characteristic

As shown in Table 2, in all routine laboratory indicators, the AUC of L% and N% for distinguishing new-onset AS from HC was higher than 0.800. To determine the predictive model based on the combination of mRNA *NAT10* and routine laboratory indicators for distinguishing new-onset AS patients from HC, and with a view to the less number of subjects, we selected mRNA *NAT10,* L% and N% for further univariable and multivariable analyses. Based on multivariate analysis, mRNA *NAT10* and N% were selected as variables for predictive model (Table 3). Based on regression coefficients, a model was built to predict new-onset AS patients from HC as follow: *P* = 0.229*N%-0.786*NAT10-12.902. P, predictive value. The value of each subject was reckoned, and a greater value would predict higher probability for new-onset AS (Fig. 5A).

**Table 2:** The performance of routine indicators

Indicator	AUC	Sensitivity (%)	Specificity (%)	Cutoff
WBC	0.676	67.31	71.15	6.810
HGB	0.698	67.31	63.46	147.5
HCT	0.640	53.85	71.15	0.4345
PLT	0.688	44.23	94.23	302.5
MPV	0.723	59.62	80.77	9.950
PDW	0.743	63.46	92.31	15.35
L	0.699	57.69	78.85	1.885
L (%)	0.839	71.15	82.69	30.2
M (%)	0.653	67.31	63.46	6.35
N	0.769	71.15	80.77	4.02
N (%)	0.857	71.15	86.54	60.95

*HCT* hematocrit, *HGB* hemoglobin, *L* lymphocyte counts, *L%* lymphocyte percentages, *M* monocyte counts, *M%* monocyte percentages, *MPV* mean platelet volume, *N* neutrophils counts, *N%* neutrophil percentages, *PCT* plateletcrit, *PDW* platelet distribution width, *PLT* platelet count, *RBC* red blood cell counts, *WBC* white blood cell counts

**Table 3:** Univariable and multivariable analysis of risk factors correalted with new-onset AS

	Univariate analysis			Multivariate analysis		
	OR	95% CI	*P*-value	OR	95% CI	*P*-value
L%	-0.209	0.745–0.884	< 0.0001			
N%	0.215	1.133–1.357	< 0.0001	0.200	1.009–1.478	0.040
NAT10	-0.651	0.331–0.822	< 0.0001	-0.790	0.262–0.786	0.005

*AS* ankylosing spondylitis, *L%* lymphocyte percentages, *NAT10* N-acetyltransferase 10, *N%* neutrophil percentages

The predictive model based on combination of mRNA *NAT10* and N% performed best in distinguishing new-onset AS patients from HC with AUC of 0.880 [95% CI, 0.816–0.944] (Fig. 5B), which was superior to mRNA *NAT10* (Fig. 4) and N% (Table 2). When the cutoff value of predictive model as -0.1876, the sensitivity was 84.62% and the specificity was 76.92%.

In addition, 45 patients were tested for HLA-B27 and 38 patients were positive. All HC were tested for HLA-B27 and were negative. Using the cutoff value for predictive model as -0.1876 and the result of the HLA-B27 test, the potential predictive value of the combination of predictive model-HLA-B27 to distinguish patients with AS from HC was explored. It was showed that the combination model of predictive model-HLA-B27 was able to effectively discriminate patients with AS from HC with a sensitivity of 92.86% (39/42), a specificity of 100.00% (52/52) and an accuracy of 96.81% (91/94) (Table 4). The potential predictive value of the combination of predictive model-HLA-B27 for AS vs. HC was superior to that of HLA-B27, which in turn had a sensitivity of 84.44% (38/45), a specificity of 100.00% (52/52) and an accuracy of 92.78% (90/97).

**Table 4:** Predictive efficiency of predictive model-HLA-B27 for AS

A, predictive model > -0.1876 or HLA-B27 positive					
Categories	Positive	Negative	sensitivity	specificity	accuracy
AS (42)	39	3	92.86% (39/42)	100% (52/52)	96.81% (91/94)
HC (52)	0	52			
B, HLA-B27 positive					
Categories	Positive	Negative	sensitivity	specificity	accuracy
AS (45)	38	7	84.44% (38/45)	100% (52/52)	92.78% (90/97)
HC (52)	0	52			

*AS* ankylosing spondylitis, *HLA-B27* human leukocyte antigen-B27, *HC* healthy control

### The predictive model based on *NAT10* and N% correlated with disease activity

**Fig. 6:**
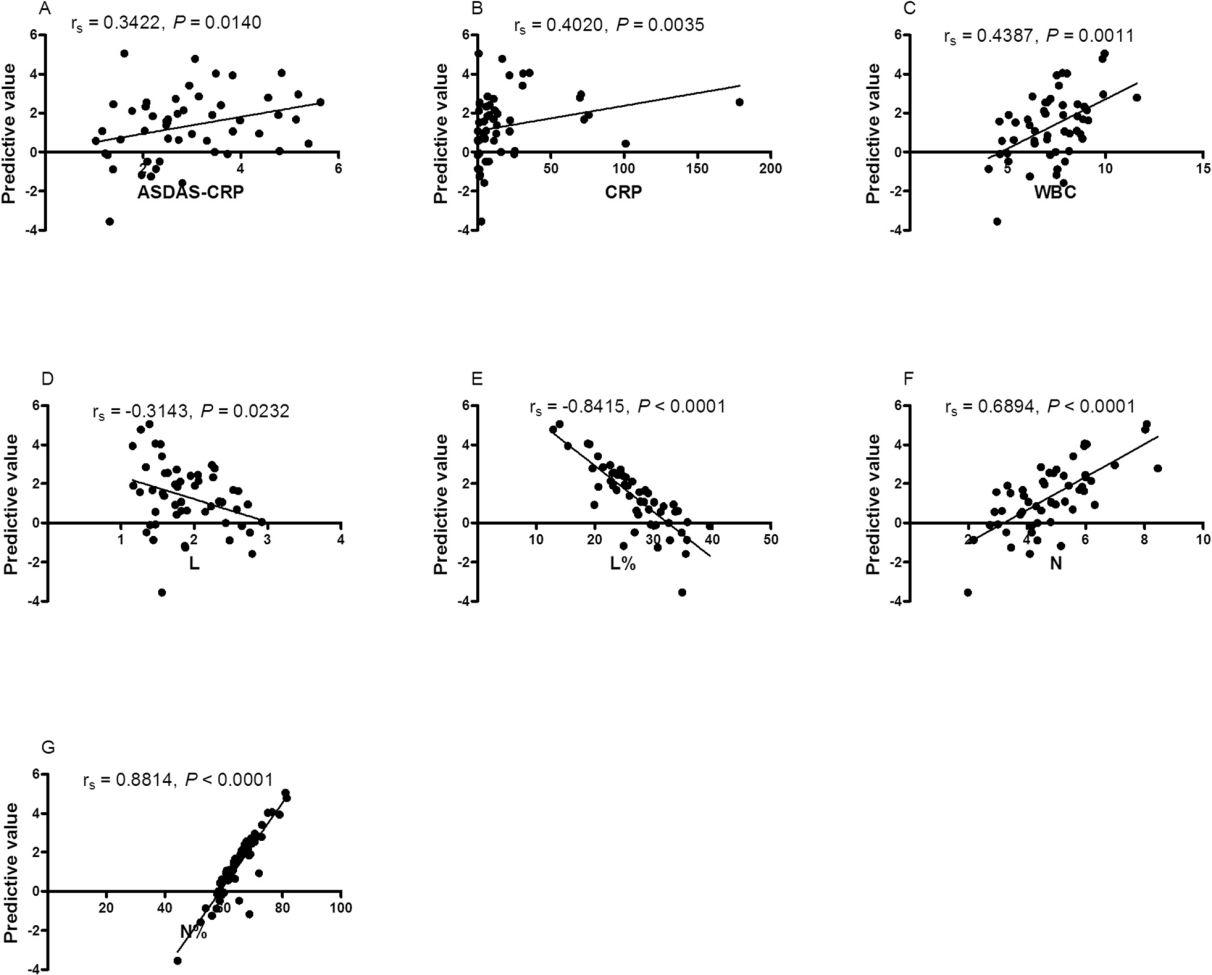
Correlation between predictive model and disease activity of AS. **A** The predictive model positively correlated with ASDAS-CRP. **B** The predictive model positively correlated with CRP. **C** The predictive model positively correlated with WBC. **D** The predictive model negatively correlated with L. **E** The predictive model negatively correlated with L%. **F** The predictive model negatively correlated with N. **G** The predictive model negatively correlated with N%. AS: ankylosing spondylitis, ASDAS: AS Disease Activity Score, CRP: C reaction protein, **L** lymphocyte counts, L%: lymphocyte percentages, **N** neutrophils counts, N%: neutrophil percentages, WBC: white blood cell counts

To explore whether the predictive model based on *NAT10* and N% could be used to assess disease activity of new-onset AS, a Spearman’s analysis was used to investigate associations between predictive value of predictive model and clinical characteristics, including ASDAS-CRP, BASDAI, BASFI, CRP, ESR, ECR, WBC, RBC, HGB, HCT, PLT, MPV, PCT, PDW, L, L%, M, M%, N, N%. More importantly, the high value of predictive model was found positively correlated with the clinical activity, as indicated by the ASDAS-CRP (r_s_ = 0.3422, *P* = 0.0140; Fig. 6A), CRP (r_s_ = 0.4020, *P* = 0.0035; Fig. 6B), WBC (r_s_ = 0.4387, *P* = 0.0011; Fig. 6C), L (r_s_ = -0.3143, *P* = 0.0232; Fig. 6D), L% (r_s_ = -0.8415, *P* < 0.0001; Fig. 6E), N (r_s_ = 0.6894, *P* < 0.0001; Fig. 6F), N% (r_s_ = 0.8814, *P* < 0.0001; Fig. 6G).

### Using NAT10 and predictive model for predicting patients with new-onset AS from RA + SLE

**Fig. 7:**
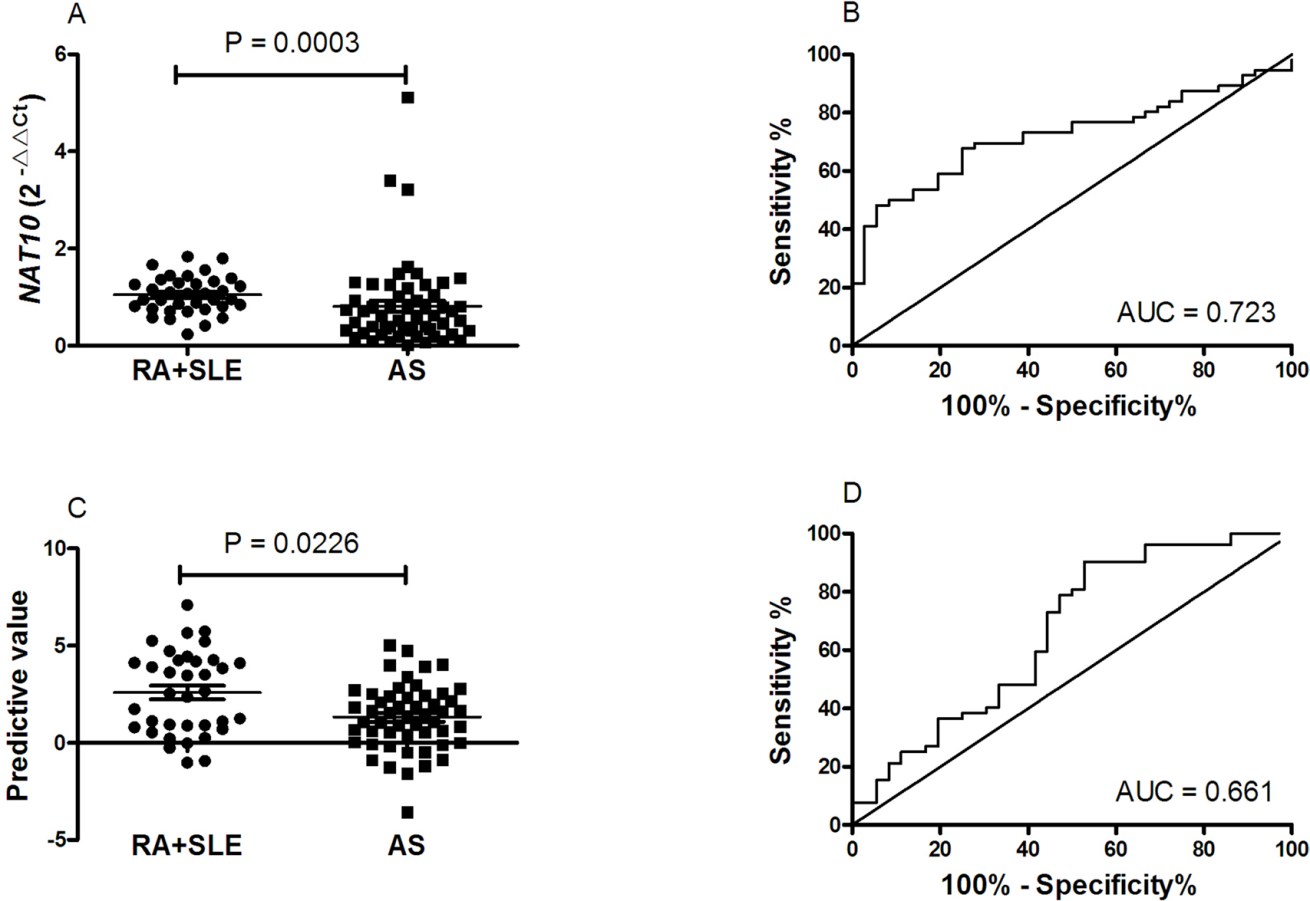
The expression of mRNA *NAT10*, the predictive value based mRNA *NAT10* and N% in RA + SLE, and their ROC analysis. **A** The expression of mRNA *NAT10* in PBMC between patients with AS and patients with RA, patients with SLE. **B** ROC analysis of the expression of mRNA *NAT10*. **C** The predictive value between patients with AS and patients with RA, patients with SLE. **D** ROC analysis of predictive model. AS: ankylosing spondylitis, NAT10: N-acetyltransferase 10, N%: neutrophil percentages, PBMC: peripheral blood mononuclear cell, RA: rheumatoid arthritis, ROC: receiver operating characteristic, SLE: systemic lupus erythematosus

Compared to RA and SLE patients, the mRNA expression level of *NAT10* in PBMC was clearly decreased in new-onset AS patients (*P* = 0.0003; Fig. 7A). Moreover, ROC curve was drawn to assess the potential predictive value of the mRNA expression level of *NAT10* in PBMC for new-onset AS, and the results showed that the AUC for distinguishing new-onset AS from RA and SLE patients was up to 0.723 (95% CI = 0.620–0.826, *P* = 0.0003; Fig. 7B), with a cutoff value of < 0.8078, a sensitivity of 67.86%, and a specificity of 75.00%.

Subsequently, the value of each RA and SLE subject was reckoned based on predictive model: *P* = 0.229*N%-0.786*NAT10-12.902, a lower value would predict higher probability for new-onset AS (Fig. 7C). And, the predictive model based on combination of mRNA *NAT10* and N% could distinguish new-onset AS patients from RA and SLE patients with AUC of 0.661 (95% CI = 0.541–0.782, *P* = 0.0104; Fig. 7D), with a cutoff value of < 3.440, a sensitivity of 90.38%, and a specificity of 47.22%.

## Discussions

To date, more and more number of RNA modifications have been reported such as N6 methyladenosine (m6A), 3 pseudouridine, 5-methylcytidine, and ac4C, which involve in mRNA stability, splicing, transport, transcription, and translation, affecting a variety of cellular and biological processes [24]. ac4C catalyzed by the NAT10 plays important roles in mRNA stability and translation [25]. NAT10 is the firstly recognized RNA acetyltransferase that participates in multiple cellular biological processes. Recently, the association of the dysregulation of NAT10 and human diseases have been discovered. It has been found that a specific NAT10 inhibitor Remodelin could be used as a potential remedy for the Hutchinson-Gilford progeria syndrome [26]. Moreover, NAT10 dysregulation is correlated with various types of tumors, infectious diseases and inflammatory diseases [8, 26–28]. However, the relationship between NAT10 dysregulation and the development of AS needs to be elucidated.

Guo et al. found that the mRNA expression of NAT10 in CD4^+^T from peripheral blood was expressed differentially in SLE patients versus HC [15]. However, little was known about the mRNA expression of NAT10 in peripheral blood in AS. The current study firstly examined mRNA expression of *NAT10* in New-Onset AS patients and healthy counterparts by RT-PCR and found that *NAT10* was significantly downregulated in PBMC from New-Onset AS patients. Our result and the study of Guo et al. [15] demonstrated the level of NAT10 in immune cell from autoimmune disease, such as AS and SLE were expressed differentially. It has been theorized that immune cell dysfunction that govern AS are linked to alteration in the level of NAT10.

In accordance with other reports showing that the expression of NAT10 in immune cell correlated with disease severity [8], our study found that the decreased mRNA expression of *NAT10* in PBMC of patients with new-onset AS negatively correlated with ASDAS-CRP and BASDIA, which indicated the activity of AS. Moreover, the decreased mRNA expression of *NAT10* in PBMC from HLA-B27 positive patients with new-onset AS negatively correlated with ASDAS-CRP, BASDIA and BASFI. These results indicated that the mRNA expression of *NAT10* in PBMC may be used as markers for disease activity in new-onset AS, especially HLA-B27 positive patients with new-onset AS.

The early correct diagnosis of AS is important in order to decrease disease burden through early intervention to reduce disability experienced by many patients. It is well-known that the diagnosis of AS depended on the patient’s history, clinical presentation, laboratory findings (HLA-B27, ESR, CRP) and diagnostic imaging findings. However, early clinical presentation and imaging changes of many HLA-B27 (-) patients are atypical and acute phase reactants of these patients are not high, and HLA-B27 is present in healthy people [29], which brings enormous diagnostic challenges to us. Recently, some review identified NAT10 as a potential target for diagnosis, therapy, and prognosis in clinical application [11]. Furthermore, evidence from Tao et al. [30] have discovered that NAT10 was a significant player in Human head and neck squamous carcinoma (HNSCC) and a promising predictive biomarker for HNSCC patients. This present study examined the potential predictive value of *NAT10* for AS and data showed that the AUC for distinguishing new-onset AS from HC was up to 0.702 (a sensitivity of 48.21%, and a specificity of 90.38%) and the AUC for distinguishing new-onset AS from RA + SLE was up to 0.723 (a sensitivity of 67.86%, and a specificity of 75.00%). The fact that *NAT10* plays a role in distinguishing new-onset AS from HC, RA + SLE and *NAT10* correlates with disease activity raises the possibility that *NAT10* might be utilized as a diagnostic, prognostic and therapeutic target for AS.

Recently, some researches testified that the combination of blood routine parameters and immunological indicators may use as predictive indicator for state, development, and prognosis of diseases [31]. And our previous study manifested that the predictive model based on m^6^A RNA-binding proteins and blood routine parameters could be used to distinguish AS patients from HC [32]. Thus, we choose mRNA *NAT10* and N% to set up a mathematical model for predicting AS according to univariable and multivariable analyses, and the predictive model based on mRNA *NAT10* and N% exhibit best value in distinguishing AS patients from HC with a AUC of 0.880, with a sensitivity of 84.62% and a specificity of 76.92%, which was superior to mRNA *NAT10* and N%. In addition, the predictive model based on mRNA *NAT10* and N% could distinguish new-onset AS patients from RA and SLE (AUC = 0.661, sensitivity = 90.38%, specificity = 47.22%). Moreover, the predictive model based on mRNA *NAT10* and N% correlated with clinical activity indicating by the ASDAS-CRP, CRP, WBC, L, L%, N, N%. These results indicated that mRNA *NAT10* and N% have synergistic role on predicting AS and evaluating activity.

Since the most commonly used biomarker in AS is HLA-B27 for diagnosis. And the combination of newer genetic biomarkers and HLA-B27 can improve the diagnostic efficiency [33]. The potential predictive value of the combination of predictive model-HLA-B27 to distinguish patients with AS from HC was explored. And, the results showed that the combination model of predictive model-HLA-B27 was able to effectively discriminate patients with AS from HC with a sensitivity of 92.86% (39/42), a specificity of 100.00% (52/52) and an accuracy of 96.81% (91/94), which was superior to that of HLA-B27 with a sensitivity of 84.44% (38/45), a specificity of 100.00% (52/52) and an accuracy of 92.78% (90/97). These data indicated that the combination of predictive model (mRNA *NAT10*-N%) and traditional biomarkers could further improve the diagnostic value.

Finally, the study had some limitations needed to state. Firstly, we only recruited some subjects from one institution, which may generate some certain risk of deviation. Secondly, our study only recruited 20 patients with RA and 16 patients with SLE as autoimmune disease control may limit value of the predictive model based on mRNA *NAT1*0 and N% in real clinical practice, due to N% also significantly rises in other inflammatory diseases excepts AS, RA, SLE. Thirdly, the level of ac4C modification and the functional role of decreased NAT10 in AS should be explored in future.

## Conclusion

The current study firstly measured the mRNA expression of *NAT10* in PBMC of AS, HC, RA, SLE and described that decreased mRNA *NAT10* in PBMC correlated with disease activity of new-onset AS. Moreover, the predictive model based on mRNA *NAT10* and N% may act as bioindicator for forecast and progression of diseases. Furthermore, the combination of predictive model (mRNA *NAT10*-N%) and B27 could further improve the diagnostic value. The precise mechanisms underlying the functions of NAT10 in AS still need to investigate. Therefore, the role of NAT10 in AS may provide insights into the pathogenesis of AS.

## Data Availability

All data generated or analysed during this study are included in this published article.

## Abbreviations

ac4C: N4-acetylcytidine
AS: Ankylosing spondylitis
ASDAS-CRP: AS Disease Activity Score-C reaction protein
AUC: Area under the curve
BASDAI: Bath AS Disease Activity Index
BASFI: Bath AS Functional Index
ESR: Erythrocyte sedimentation rate
HC: Healthy controls
HCT: Hematocrit
HGB: Hemoglobin
HLA-B27: Human leukocyte antigen-B27
HNSCC: Head and neck squamous carcinoma
L: Lymphocyte counts
L%: Lymphocyte percentages
M: Monocyte counts
M%: Monocyte percentages
MPV: Mean platelet volume
N: Neutrophil counts
N%: Neutrophil percentages
NAT10: N-acetyltransferase 10
PCT: Plateletcrit
PBMC: Peripheral blood mononuclear cell
PDW: Platelet distribution width
PLT: Platelet count
RA: Rheumatoid arthritis
RBC: Red blood cell counts
ROC: Receiver operating characteristic
SLE: Systemic lupus erythematosus
WBC: White blood cell counts

## References

[1] Mauro D, Thomas R, Guggino G, Lories R, Brown MA, Ciccia F (2021). Ankylosing spondylitis: An autoimmune or autoinflammatory disease?. Nat Rev Rheumatol.

[2] Fang Y, Liu J (2023). Novel regulatory role of non-coding RNAs in ankylosing spondylitis. Front Immunol.

[3] Ward MM, Deodhar A, Gensler LS, Dubreuil M, Yu D, Khan MA, Haroon N, Borenstein D, Wang R, Biehl A, Fang MA, Louie G, Majithia V, Ng B, Bigham R, Pianin M, Shah AA, Sullivan N, Turgunbaev M, Oristaglio J, Turner A, Maksymowych WP, Caplan L (2019). 2019 Update of the American College of Rheumatology/Spondylitis Association of America/Spondyloarthritis Research and Treatment Network recommendations for the treatment of ankylosing spondylitis and nonradiographic axial spondyloarthritis. Arthritis Rheumatol.

[4] Maksymowych WP (2019). Biomarkers for diagnosis of axial spondyloarthritis, disease activity, prognosis, and prediction of response to therapy. Front Immunol.

[5] Dominissini D, Rechavi G (2018). N(4)-Acetylation of Cytidine in mRNA by NAT10 Regulates Stability and Translation. Cell.

[6] Zi J, Han Q, Gu S, McGrath M, Kane S, Song C (2020). Targeting NAT10 induces apoptosis associated with enhancing endoplasmic reticulum stress in acute myeloid leukemia cells. Front Oncol.

[7] Liu X, Cai S, Zhang C, Liu Z, Luo J, Xing B (2018). Deacetylation of NAT10 by Sirt1 promotes the transition from rRNA biogenesis to autophagy upon energy stress. Nucleic Acids Res.

[8] Zhang H, Chen Z, Zhou J, Gu J, Wu H, Jiang Y (2022). NAT10 regulates neutrophil pyroptosis in sepsis via acetylating ULK1 RNA and activating STING pathway. Commun Biol.

[9] Li Q, Liu X, Jin K, Lu M, Zhang C, Du X (2017). NAT10 is upregulated in hepatocellular carcinoma and enhances mutant p53 activity. BMC Cancer.

[10] Zhang Y, Jing Y, Wang Y, Tang J, Zhu X, Jin W (2021). NAT10 promotes gastric cancer metastasis via N4-acetylated COL5A1. Signal Transduct Target Ther.

[11] Liang P, Hu R, Liu Z, Miao M, Jiang H, Li C (2020). NAT10 upregulation indicates a poor prognosis in acute myeloid leukemia. Curr Probl Cancer.

[12] Xie L, Zhong X, Cao W, Liu J, Zu X, Chen L (2023). Mechanisms of NAT10 as ac4C writer in diseases. Mol Ther Nucleic Acids.

[13] Tsai K, Jaguva Vasudevan AA, Martinez Campos C, Emery A, Swanstrom R, Cullen BR (2020). Acetylation of cytidine residues boosts HIV-1 gene expression by increasing viral RNA stability. Cell Host Microbe.

[14] Furuse Y (2021). RNA modifications in genomic RNA of Influenza A Virus and the relationship between RNA modifications and viral infection. Int J Mol Sci.

[15] Guo G, Shi X, Wang H, Ye L, Tong X, Yan K (2020). Epitranscriptomic N4-acetylcytidine profiling in CD4+ T cells of systemic lupus erythematosus. Front Cell Dev Biol.

[16] Duan J, Zhang Q, Hu X, Lu D, Yu W, Bai H (2019). N(4)-Acetylcytidine is Required for Sustained Nlrp3 Inflammasome Activation Via Hmgb1 Pathway in Microglia. Cell Signal.

[17] van der Linden S, Valkenburg HA, Cats A (1984). Evaluation of diagnostic criteria for ankylosing spondylitis. A proposal for modification of the New York criteria. Arthritis Rheum..

[18] Arnett FC, Edworthy SM, Bloch DA, McShane DJ, Fries JF, Cooper NS (1988). The American Rheumatism Association 1987 revised criteria for the classification of rheumatoid arthritis. Arthritis Rheum.

[19] Hochberg MC (1997). Updating the American College of Rheumatology revised criteria for the classification of systemic lupus erythematosus. Arthritis Rheum.

[20] Luo Q, Zhang L, Li X, Fu B, Guo Y, Huang Z (2019). Identification of circular RNA hsa_circ_0044235 and hsa_circ_0068367 as novel biomarkers for systemic lupus erythematosus. Int J Mol Med.

[21] Livak KJ, Schmittgen TD (2001). Analysis of relative gene expression data using realtime quantitative PCR and the 2(−Delta Delta C(T)) method. Methods.

[22] Machado P, Landewé R, Lie E, Kvien TK, Braun J, Baker D (2011). Assessment of spondyloarthritis international society: ankylosing spondylitis disease activity score (ASDAS): defining cut-off values for disease activity states and improvement scores. Ann Rheum Dis.

[23] Garrett S, Jenkinson T, Kennedy LG, Whitelock H, Gaisford P, Calin A (1994). A new approach to defining disease status in ankylosing spondylitis: the bath ankylosing spondylitis disease activity index. J Rheumatol.

[24] Roundtree IA, Evans ME, Pan T, He C (2017). Dynamic RNA modifications in gene expression regulation. Cell.

[25] Arango D, Sturgill D, Alhusaini N, Dillman AA, Sweet TJ, Hanson G (2018). Acetylation of cytidine in mRNA promotes translation efficiency. Cell.

[26] Larrieu D, Britton S, Demir M, Rodriguez R, Jackson SP (2014). Chemical inhibition of NAT10 corrects defects of laminopathic cells. Science.

[27] Wu J, Zhu H, Wu J, Chen W, Guan X (2018). Inhibition of N-acetyltransferase 10 using remodelin attenuates doxorubicin resistance by reversing the epithelial-mesenchymal transition in breast cancer. Am J Transl Res.

[28] Tsai K, Jaguva Vasudevan AA, Martinez Campos C, Emery A, Swanstrom R, Cullen BR (2020). Acetylation of cytidine residues boosts HIV-1 gene expression by increasing viral RNA stability. Cell Host Microbe.

[29] Elyan M, Khan MA (2006). Diagnosing ankylosing spondylitis. J Rheumatol Suppl.

[30] Tao W, Tian G, Xu S, Li J, Zhang Z, Li J (2021). NAT10 as a potential prognostic biomarker and therapeutic target for HNSCC. Cancer Cell Int.

[31] Tang G, Tong S, Yuan X, Lin Q, Luo Y, Song H (2021). Using Routine Laboratory Markers and Immunological Indicators for Predicting Pneumocystis jiroveci Pneumonia in Immunocompromised Patients. Front Immunol.

[32] Luo Q, Guo Y, Xiao Q, Fu B, Zhang L, Guo Y (2022). Expression and Clinical Significance of the m6A RNA-Binding Proteins YTHDF2 in Peripheral Blood Mononuclear Cells From New-Onset Ankylosing Spondylitis. Front Med (Lausanne).

[33] Diaconu AD, Ceasovschih A, Șorodoc V, Pomîrleanu C, Lionte C, Șorodoc L, Ancuța C (2022). Practical Significance of Biomarkers in Axial Spondyloarthritis: Updates on Diagnosis, Disease Activity, and Prognosis. Int J Mol Sci.

